# Diagnostic accuracy of loop-mediated isothermal amplification (LAMP) for screening patients with imported malaria in a non-endemic setting

**DOI:** 10.1051/parasite/2017054

**Published:** 2017-12-18

**Authors:** Camille Ponce, Flora Kaczorowski, Thomas Perpoint, Patrick Miailhes, Alain Sigal, Etienne Javouhey, Yves Gillet, Laurent Jacquin, Marion Douplat, Karim Tazarourte, Véronique Potinet, Bruno Simon, Adeline Lavoignat, Guillaume Bonnot, Fatimata Sow, Anne-Lise Bienvenu, Stéphane Picot

**Affiliations:** 1 Institute of Parasitology and Medical Mycology, Hôpital de la Croix-Rousse, Hospices Civils de Lyon, 69004 Lyon France; 2 Service des Maladies Infectieuses et Tropicales, Hôpital de la Croix-Rousse, Hospices Civils de Lyon, 69004 Lyon France; 3 Service d'accueil des Urgences, Hôpital de la Croix-Rousse, Hospices Civils de Lyon, 69004 Lyon France; 4 Service d'accueil des Urgences Pédiatriques, Hôpital Femme-Mère-Enfant, Hospices Civils de Lyon, 69677 Bron France; 5 Service d'accueil des urgences, Hôpital Edouard Herriot, Hospices Civils de Lyon, 69003 Lyon France; 6 Service d'accueil des urgences, Hôpital Lyon Sud, Hospices Civils de Lyon, Hôpital Lyon Sud, 69310 Pierre-Bénite France; 7 Service des urgences/SAMU 69, Hospices Civils de Lyon, Lyon, 69003 France; 8 Univ. Lyon, Université Claude Bernard Lyon 1, HESPER EA 7425, 69008 Lyon France; 9 Malaria Research Unit, SMITh, ICBMS, UMR 5246 CNRS-INSA-CPE-University Lyon1, 69100 Villeurbanne France; 10 Laboratoire d'Hématologie, Hôpital de la Croix-Rousse, Hospices Civils de Lyon, 69004 Lyon France

**Keywords:** Malaria, diagnosis, Plasmodium, isothermal amplification, LAMP, microscopy

## Abstract

**Background**: Sensitive and easy-to-perform methods for the diagnosis of malaria are not yet available. Improving the limit of detection and following the requirements for certification are issues to be addressed in both endemic and non-endemic settings. The aim of this study was to test whether loop-mediated isothermal amplification of DNA (LAMP) may be an alternative to microscopy or real-time PCR for the screening of imported malaria cases in non-endemic area. **Results**: 310 blood samples associated with 829 suspected cases of imported malaria were tested during a one year period. Microscopy (thin and thick stained blood slides, reference standard) was used for the diagnosis. Real-time PCR was used as a standard of truth, and LAMP (Meridian Malaria Plus) was used as an index test in a prospective study conducted following the Standards for Reporting Diagnosis Accuracy Studies. In the 83 positive samples, species identification was *P. falciparum* (n = 66), *P. ovale* (n = 9), *P. vivax* (n = 3) *P. malariae* (n = 3) and 2 co-infections with *P. falciparum* + *P.malariae*. Using LAMP methods, 93 samples gave positive results, including 4 false-positives. Sensitivity, specificity, positive predictive value and negative predictive value for LAMP tests were 100%, 98.13%, 95.51%, and 100% compared to PCR. **Conclusion**: High negative predictive value, and limit of detection suggest that LAMP can be used for screening of imported malaria cases in non-endemic countries when expert microscopists are not immediately available. However, the rare occurrence of non-valid results and the need for species identification and quantification of positive samples preclude the use of LAMP as a single reference method.

## Abstract (fr)

**Contexte** : Des méthodes sensibles et faciles pour le diagnostic du paludisme sont encore attendues. Améliorer la limite de détection et répondre aux besoins de la certification sont des questions auxquelles il faut répondre tant en zone endémique que non-endémique. L'objectif de cette étude était de tester si la méthode d'amplification isothermique d'ADN en boucle (LAMP) peut être une alternative à la microscopie ou à la PCR temps réel pour le dépistage des cas de paludisme d'importation en zone non-endémique. **Résultats** : 310 échantillons de sang provenant de 829 suspicions de paludisme importé ont été testés pendant l'année de l'étude. La microscopie (frottis sanguins et gouttes épaisses colorées, standard de référence) a été utilisée pour le diagnostic. La PCR temps réel a été utilisée comme standard de vérité, et la LAMP (Meridian Malaria Plus) a été utilisée en méthode index dans une étude prospective conduite selon les standards pour rapporter les études d'efficacité diagnostique. Parmi les 83 échantillons positifs, l'identification des espèces était *P. falciparum* (n = 66), *P. ovale* (n = 9), *P. vivax* (n = 3) *P. malariae* (n = 3) et 2 coïnfections *P. falciparum* + *P. malariae*. La méthode LAMP a donné 93 échantillons positifs, dont 4 faux positifs. La sensitivité, la spécificité, la valeur prédictive positive et la valeur prédictive négative pour le LAMP étaient 100 %, 98.13 %, 95.51 %, 100 % comparées à la PCR. **Conclusion** : Une valeur prédictive négative élevée et la limite de détection suggèrent que le LAMP peut être utilisé pour dépister les cas de paludisme d'importation en zone non-endémique lorsque des microscopistes experts ne sont pas immédiatement disponibles. Cependant, la rare possibilité de résultats non valides et le besoin d'une identification d'espèce et d'une quantification empêchent que la méthode LAMP soit utilisée comme seule méthode de référence.

## Introduction

The global malaria burden has been considerably reduced during the last decade thanks to insecticide-treated bed nets, rapid diagnosis tests (RDTs) and highly effective antimalarial treatment. But malaria remains a substantial global health problem [29] and elimination has been pointed out as a reachable goal in the next few decades [16]. One of the major issues to be addressed on the way toward malaria elimination is the development of a highly sensitive, reliable and easy-to-perform method for the point-of-care diagnosis of malaria [3].

Biological diagnosis of malaria can be conducted using light microscopy, RDTs, PCR, or a combination of these methods. Microscopic examination of Giemsa-stained thick or thin smears was the reference standard all over the world for more than a century. But microscopy is recommended for malaria when its quality can be maintained and strong expertise is available. Unfortunately, in endemic and non-endemic countries, expert malaria microscopists are uncommon in non-specialized clinical laboratories and impaired microscopy-based diagnosis, especially for non-*falciparum* species, can be suspected [1]. The limit of detection (LOD) may vary substantially depending on the experience and training of microscopists, with a range of 5 to 100 parasites/μL [31]. The requirements for certification of biological diagnosis have increased the need for efficient and standardized methods that are easy to perform for end-users and that are reliable when microscopic expertise is not immediately available.

Since the early 1990s, the advent of RDTs for malaria [18] has profoundly increased the availability of reliable diagnosis in remote areas. However, the overall sensitivity of most of the RDTs available on the market is high for parasitemia over 200 parasites/μL, but substantially decreased for lower parasite burdens [30]. The molecular detection of malaria parasites has definitively proved its capacity to detect low parasitemia with LOD of 0.05 parasites/μL or less, depending on many technical factors, including the volume of blood sampled. However, this method is still limited to well-equipped centers mainly outside remote endemic areas and this causes delays that are in conflict with the need for rapid diagnosis to promptly initiate documented treatment of malaria cases.

The recent development of loop-mediated isothermal amplification of DNA (LAMP) in 2000 [17] and its initial application to malaria diagnosis in 2006 [23] mean that it may be a suitable tool for diagnosis in endemic and non-endemic areas [19,22].

LAMP is an isothermal molecular method using a DNA polymerase from *Bacillus stearothermophilus* which has strand displacement activity leading to DNA auto-cycling without temperature changes. The different tests available, mostly targeting the mitochondrial genome of the *Plasmodium* parasite, required a set of six different primers.

The sensitivity and specificity of LAMP have come close to those of PCR [28]. The corresponding naked eye reading makes it a good candidate for field use, although some improvements are still needed. Numerous studies have attempted to address certain weak points by eliminating the need for electricity [14,26], optimizing high throughput [3,4,6], or allowing measurement of parasitemia [12].

The primary objective of this study was to test a LAMP method commercially available (Illumigene Malaria Plus test, Meridian Bioscience Inc., Cincinnati, OH, USA) in daily diagnostic conditions of imported malaria cases in a specialized laboratory of a teaching hospital in a non-endemic area. The secondary objective was to evaluate its relevance as a screening test to be used when expert malaria microscopists are not immediately available. We chose to compare the index test (LAMP), to a reference standard (light microscopy) and to a standard of truth (real-time PCR).

Among a one-year consecutive series of 829 blood samples collected for malaria diagnosis in our institution, 310 fresh blood samples from patients suspected of imported malaria were included. LAMP tests were conducted rapidly after completion of standard diagnostic procedures including microscopic examination of thick and thin blood smears and RDT. Real-time PCR was performed from frozen samples after the initial diagnosis. The limit of detection of the Illumigene test was determined using two clones of *Plasmodium falciparum* cultivated *in vitro*.

## Materials and methods

### Ethical clearance

This research involved anonymized records and datasets where it is not possible to identify individuals from the information provided. De-identification and removing of protected health information from clinical narratives were performed according to the European Textbook on Ethics in research (http://ec.europa.eu/research/swafs/pdf/pub_archive/textbook-on-ethics-report_en.pdf). Data used in this study were collected for the routine diagnosis and clinical management of patients at Lyon teaching hospital, and no additional interventions involving patients were planned for research purposes.

### Study design

This was a prospective study conducted according to the Standards for Reporting Diagnosis Accuracy Studies (STARD 2015) [8].

### Participants

**Figure 1:**
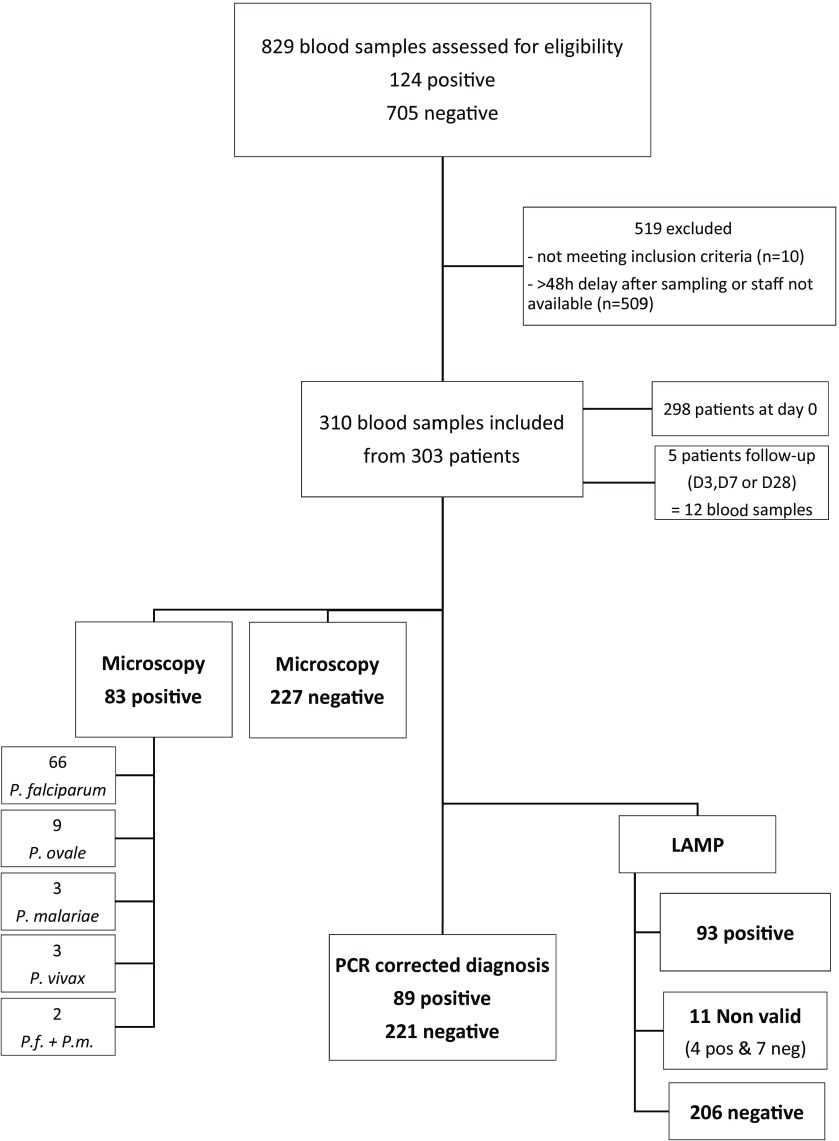
Study flow chart.

Blood samples were collected from all patients suspected of malaria infection at Lyon University Hospital. Eligibility criteria were suspicion of malaria based on fever and history of travel to malaria endemic areas, or history of treated or untreated malaria. Patients were assessed for eligibility and included during a one-year period (June 2016 to June 2017) (Figure 1). Light microscopy (examination of thin and thick blood stains) was used as a reference diagnosis to classify blood as positive or negative for *Plasmodium*. The final diagnosis was based only on the results of the reference tests. Lamp tests (index test) were conducted from fresh blood samples (stored at + 4 °C for less than 48 hours) and real-time PCR tests (standard of truth) were conducted from aliquots of samples frozen (–20 °C) after collection. Neither real-time PCR nor LAMP tests were used for clinical malaria diagnosis.

### Patient blood samples

Venous blood samples were collected on EDTA vacutainer tubes and immediately transferred to the laboratory at room temperature. For each blood sample, thin and thick blood stained smears (Diff-Quick and Giemsa stains, respectively) and RDT (Vikia Malaria, BioMérieux, France) [10] were performed in compliance with the recommendations of French guidelines for malaria diagnosis, and following certification requirements of the National Certification Program. Microscopy was performed by two independent trained and certified biologists and discrepancies were resolved by a senior microscopist. The protocol was implemented in accordance with standard operating procedures and in compliance with good clinical laboratory practices. Proficiency testing and External Quality Assessment for microscopy were used once a week and bi-monthly, respectively. Parasitemia was recorded as the number of red blood cells (RBCs) infected with asexual parasites compared to non-infected RBCs. Malaria diagnosis was considered to be negative if no parasites were found in 100 microscopic fields of 200 red blood cells for thin smears and 25 microscopic high power fields of thick smears [24]. PCR confirmation was later obtained using real-time PCR, as previously described [15]. None of the samples were repeat specimens.

### *In vitro* parasite culture

3D7 (cloned from NF54 isolated at Schiphol airport Amsterdam, of unknown origin, probably Africa) and W2 (cloned in 1981 from mixed culture Sierra Leone SL I / CDC  / Indochina III / CDC) clones of *Plasmodium falciparum* [20] obtained from the Malaria Research and Reference Reagent Resource Center (MR4) were used. These clones were maintained in continuous culture in an incubator at 37 °C, in a humid atmosphere with 5% CO_2_. Parasites were grown on RBC rhesus O+, at a hematocrit of 5% in RPMI 1640 added to 25 mM Hepes, 50 μg/mL gentamycin, 0.1 mM hypoxanthine, 20 mM NaHCO3 and 0.5% AlbuMax II, in culture flasks (25 cm^2^). Cultivated asynchronous parasites were serially diluted in non-infected blood from the biobank (from 100 parasites/μL to 0.0001 parasites/μL) after washing out the culture medium to reduce the amount of soluble parasite DNA accumulated during the culture. Non-infected human blood was used as a negative control.

### LAMP test

Whole blood specimens were processed using size exclusion chromatography by gravity flow to separate and purify nucleic acids. Sample effluent collected after the addition of the last buffer contains nucleic acids. The Illumigene Malaria PLUS DNA amplification assays were used (Meridian Bioscience Inc., Cincinnati, OH, USA) according to the manufacturer's specifications. This assay targets a 214 base pair sequence of a *Plasmodium* sp. mitochondrial DNA noncoding region that is conserved across *Plasmodium falciparum*, *Plasmodium vivax*, *Plasmodium ovale*, *Plasmodium malariae*, and *Plasmodium knowlesi*. The exact sequences of the 6 primers are not available from Meridian Inc. as part of intellectual property rights.

A by-product of amplification is magnesium pyrophosphate, which forms a white precipitate leading to a turbid reaction solution. Reaction solution absorbance characteristics are monitored by the Meridian illumipro-10 Incubator/Reader.

The Illumigene Malaria Test Device contains one lyophilized amplification reagent bead in each of two chambers: a test chamber with *Plasmodium sp*-specific primers and a control chamber with human mitochondrial DNA specific primers as internal controls for the assay.

The reader calculates the change in light transmission between the beginning and the end of the test (Sf: Si) and compares the ratio to a fixed cut-off value of 70%. If the Sf:Si ratio is less than 70%, the sample is reported positive. A different cut-off value (85%) for the control chamber is used to determine the validity of the test (absence of amplification or presence of inhibitor). If the control test is invalid, the sample data (positive or negative) is not reported and thus not available.

### Real-time PCR

DNA extraction was performed using the QIAamp DNA Mini kit (Qiagen, Hilden, Germany) according to the manufacturer's instructions, starting with 200 μL of the parasite cultures. DNA was eluted with 50 μL of elution buffer AE. Real-time PCR, using LightCycler technology (Roche Molecular Biochemicals, Mannheim, Germany) was performed with fluorescent SYBR Green I dye, as previously described [15]. The extracted DNA (5 μL) was added to 15 μL of reaction mixture containing 3 mM of MgCl2, 0.5 μM of each primer, and 2 μL of LC Fast Start DNA Master SYBR-Green I buffer. Conditions for cycling were 95 °C for 10 minutes, followed by 36 cycles at 95 °C for 10 seconds, 58 °C for 10 seconds and 72 °C for 30 seconds, with monitoring of fluorescence at the end of the elongation steps. PCR product identity was confirmed with melting curves analysis. The melting program consisted of 1 cycle at 95 °C for 2 seconds, 55 °C for 20 seconds, and heating at 95 °C. A red blood cell control and a non-template control were included to test the amplification specificity and to allow detection of possible contamination of the PCR mixture, respectively. This method was routinely used for malaria diagnosis and clinically validated [15].

### Data analysis

Data were pooled into double entry tables to compute the sensitivity, specificity, and positive and negative predictive values of each test, including 95% confidence intervals using MEDCALP statistical software available at https://www.medcalc.org/calc/diagnostic_test.php.

## Results

### Blood samples from patients

829 blood samples for malaria diagnosis were received during the one-year period. Most of the patients were adults presenting non-severe malaria (n = 721). None of the patients included had a fatal outcome. Roughly two thirds of these samples (519) were excluded from the study mainly due to unavailability of staff performing the LAMP test during the first 48 hours after blood sampling, since only three biologists out of six involved in malaria diagnosis were trained and certified to perform LAMP tests. This did not result in recruitment bias since availability was randomly based on the on-duty agenda of the team. Three hundred and ten blood samples from patients were included in the study. Clinical information and reference standard results were available to the performers during the LAMP test. LAMP results were later made available to the assessors of the reference test, but never used for the diagnosis of patients.

### Performance of the tests

The absence of *Plasmodium* was determined from 227 samples using microscopic thick and thin blood smears examination. Among the 83 positive samples, species identification showed *P. falciparum* (n = 66; 79.5%), *P. ovale* (n = 9; 10.9%), *P. vivax* (n = 3; 3.6%), *P. malariae* (n = 3; 3.6%) and 2 co-infections with *P. falciparum* and *P. malariae* (2.4%). The high rate of *Plasmodium* *falciparum* versus non-*falciparum* species was expected since more than 80% of patients attending Lyon Hospital were infected in Africa, as observed for several decades.

Using real-time PCR, 89 positive samples were detected. The 6 real-time PCR corrected positive diagnoses were samples from patient follow-up after treatment at days 7 and 28.

Using LAMP methods, 93 samples gave positive results, including 4 false-positives, according to real-time PCR considered as the standard of truth. These false-positive test results were considered to be due to DNA contamination during sample processing, as demonstrated by further tests. Using the LAMP Illumigen reader, eleven samples provided “invalid” results. While these tests were repeated once again, it was not possible to obtain a result to classify these samples with the LAMP method for unknown reasons. These samples were excluded from further analysis (Figure 1).

Performances of the method were calculated from 299 samples (excluding the 11 invalid results). Sensitivity and negative predictive values were 100% compared to microscopy and to real-time PCR (Table 1). Specificity and positive predictive value were slightly better compared to real-time PCR than to microscopy tests: 93.64 (CI95%: 89.55 to 96.48) / 98.13 (CI95%: 95.28 to 99.49) and 84.95 (CI95%: 77.27 to 90.35) / 95.51 (CI95%: 88.95 to 98.25), respectively.

**Table 1:** Performance comparison between LAMP and microscopy or real-time PCR. Sensitivity, specificity, positive predictive value and negative predictive value were calculated based on 299 samples, excluding the 11 invalid results from LAMP. The number of samples used for microscopy are 79 positive (83–4 invalid excluded) and 220 negative (227–7 invalid excluded). The number of samples used for PCR are 85 positive (89–4 invalid excluded) and 214 negative (221–7 invalid excluded).

		Microscopy			Real-time PCR		
			
		+	−	total	+	−	total
LAMP	+	79	14	93	85	4	89
	−	0	206	206	0	210	210
		79	220	299	85	214	299

	Versus microscopy		Versus real-time PCR	
		
LAMP performance	%	95% confidence interval (%)	%	95% confidence interval (%)
Sensitivity	100	95.55 to 100	100	95.75 to 100
Specificity	93.64	89.55 to 96.48	98.13	95.28 to 99.49
Positive predictive value	84.95	77.27 to 90.35	95.51	88.95 to 98.25
Negative predictive value	100	n.a.	100	n.a.
Positive likelihood ratio	15.7	n.a.	53.5	n.a.

While the sample size was limited, the four main *Plasmodium* species (*P. falciparum*, *P.* *ovale*, *P. malariae* and *P. vivax*) were tested with parasitemia ranging from 450 to 315.000 parasites/μL. The LAMP method gave positive results for most of these species, except one *P. vivax* sample. We did not have epidemiological suspicion of *Plasmodium knowlesi* infection in our panel (patient contamination in South-East Asia and non-*falciparum* microscopic evidence), but all the parasites identified as *Plasmodium malariae* were confirmed by species-specific real-time PCR. We did not test *P. knowlesi* confirmed samples and pure gametocytes samples.

Six blood samples, considered as negative with microscopic methods and collected at day 28 ± 3 after treatment of patients presenting *P. falciparum* malaria were positive using Lamp, raising the issue of high sensitivity drawback for clinical diagnosis during the follow-up of a treated patient, opposed to the advantages of high sensitivity in pre-elimination settings. These patients were apyretic and asymptomatic, and slides remained classified as negative after control. The real-time PCRs were positive for these samples. As a result, these patients presented either sub-microscopic parasitemia or residual circulating DNA. No relapse was recorded for these patients at least two months after the end of 28-day follow-up.

### Parasite clones

Serial dilutions of parasites from cultivated clones were inoculated to controlled negative human blood in order to obtain final parasite concentrations from 100 parasites/μL to 0.0001 parasites/μL (Table 2). LAMP tests were conducted in triplicate for each dilution and gave positive results up to a threshold of 0.1 parasites/μL for both parasite clones, demonstrating a higher sensitivity of this method than that reported by the manufacturer (0.25 parasite/μL, 3D7 clone). It should be noted that clones were washed three times before dilution in order to avoid contamination with soluble parasite DNA accumulated during the culture process. While time limits (below 2 minutes) for processing samples before starting the run were strictly respected, we faced rare occurrence of invalid results with the highest concentration of some samples. A limit of detection of 0.01 parasites/μL was obtained for both clones using real-time PCR with the same batch of dilution and the same volume of samples before DNA extraction.

**Table 2:** Limit of detection of the Illumigen Malaria plus LAMP method. Serial dilutions of *Plasmodium falciparum in vitro* cultivated clones 3D7 and W2 were tested using light microscopy (Giemsa stained thin blood smears), real-time PCR and LAMP Illumigen malaria. (inv. = invalid)

Plasmodium falciparum clones	Parasitemia (dilutions from thin blood smears)		Real-time PCR (35 cycles)	LAMP Illumigen Malaria (Meridian)			
			
	%	Parasites /μL		Repeated measures			LAMP interpretation
3D7	0.002	100	Positive	inv.	+	+	Positive
–	0.0002	10	Positive	+	+	+	Positive
–	0.00002	1	Positive	+	inv.	+	Positive
–	0.000002	0.1	Positive	+	−	+	Positive
–	0.0000002	0.01	Positive	−	−	−	Negative
–	0.00000002	0.001	Negative	−	−	−	Negative
–	0.00000002	0.0001	Negative	−	−	−	Negative

W2	0.002	100	Positive	+	+	inv.	Positive
–	0.0002	10	Positive	+	+	inv.	Positive
–	0.00002	1	Positive	+	+	+	Positive
–	0.000002	0.1	Positive	+	+	+	Positive
–	0.000002	0.01	Positive	−	−	−	Negative
–	0.0000002	0.001	Negative	−	−	−	Negative
–	0.00000002	0.0001	Negative	−	−	−	Negative

## Discussion

The goal of this study was to evaluate over a one-year period, the advantages of a commercially available LAMP diagnostic test for malaria compared to light microscopy and real-time PCR, using standard laboratory conditions on a daily basis in a non-endemic area hospital. A secondary objective was to evaluate the potential of this method to be used in a larger study to test its relevance for malaria screening in clinical laboratories on call conditions during the night. Two different versions of the test are available from the provider, with different sensitivities. We only tested the highest sensitivity kit [13], requiring more analytical steps but expected to have a lower detection threshold, since one of the objectives was to evaluate the potential of this method to be used as a first-line diagnostic test when expert microscopist are not available. Sensitivity and specificity of LAMP compared to microscopy have already been shown to be 98% and 97%, respectively from a meta-analysis of molecular methods for malaria diagnosis [24]. A recent study from Canada [25] has shown a sensitivity of 100.0% [95.1- 100.0%] and specificity of 91.5% [81.3 − 97.2%] versus reference microscopy. Similar remarkable results were obtained in this study (sensitivity of 100% [95.55 to 100%] and specificity of 93.64% [89.55 to 96.48%] versus microscopy. The same commercially available test was evaluated in endemic areas [13], showing sensitivity of 97.2% [92.6 to 99.1%] and specificity of 87.7% [76.6 to 94.2%] versus PET-PCR, while results presented here showed sensitivity of 100% [95.75% to 100%] and specificity of 98.13% [95.28% to 99.49%] compared to real-time PCR. Several in-house methodological improvements of the LAMP method have been tested recently in different conditions, precluding significant comparison with a commercially available test.

A systematic review and meta-analysis of loop-mediated isothermal amplification for malaria diagnosis is in preparation to compare the results from all available studies (Prospero  2017 CRD42017075186: http://www.crd.york.ac.uk/PROSPERO/display_record.php?ID=CRD42017075186).

Moreover, we tested the limit of detection of the Meridian Malaria Plus test with serial dilutions of cultivated clones synchronized at young trophozoite stages just before dilutions (3D7: 95% young trophozoites; W2: 75% young trophozoites). Culture samples were washed out three times before mixing with negative blood from the blood bank to drastically reduce the amount of free DNA. We observed a limit of detection of 0.1 parasites/μL, which is 2.5 times lower than the LOD provided by the manufacturer, and 10 times lower than the LOD of the real-time PCR method developed a decade ago in the laboratory. The LOD indicated by the manufacturer was obtained from a “theoretical probability” of obtaining positive results, as indicated in the handbook. It should be mentioned that in the same document, the LOD for *Plasmodium vivax* is indicated as 0.063 parasites/μL, which needs to be documented further. We did not test the LOD for non-*falciparum* species since cultures are not available, and dilutions of blood from infected patients should be associated with contamination by circulating parasite DNA, leading to false results.

In our laboratory, the method was easy to perform without requiring more than one hour training, and provided reliable data. It was simple to test one to four samples in the same run, including systematically a negative control, and to perform two runs in parallel on the same machine, when needed. The machine is small, linked to a dedicated printer, and did not require any connection except a power supply. The sample preparation required 4 steps of 2 minutes, and the amplification run was completed within 40 minutes, leading to a time-to-result period of less than one hour. Taken together, the practicality of the machine and the test are good, but should be limited to laboratory settings, including those in endemic areas, with a sufficient level of equipment and biological skills. The tested LAMP method (Illumigene Malaria Plus test, Meridian Bioscience Inc., Cincinnati, OH, USA) is not suitable for point-of-care use since it requires blood sample preparation and measures against DNA cross-contamination. However, the LAMP methodology could probably be adapted to field conditions, provided simplification of the process could be obtained, without decreasing sensitivity and negative predictive value [5,6,26].

The most disappointing situation is the “invalid” code, without the possibility of understanding the reason of this failure (high parasitemia, DNA amplification inhibitor, delay in the extraction process). This invalid code meant that the biologist had to perform other diagnostic tests (microscopic, RDT, PCR), after having spent one hour on the method.

One drawback of this method is that the result is restricted to a positive/negative outcome, with no information on species identification and parasite burdens. Thus, this method is not eligible as a single diagnostic tool in non-endemic areas where all *Plasmodium* species can be expected from imported cases. However, in many African field areas, the high majority of transmitted parasites are *Plasmodium falciparum*, with a limited risk of other species infection (mainly *P. ovale* and *P. malariae*, and rare occurrence of *P. vivax* in sub-Saharan Africa). Considering that the main issue to be addressed is the detection of the lowest parasitemia, the lack of information on species identification and parasite burden appears not to be a definitive limitation for this test. However, other LAMP tests have been developed to discriminate *Plasmodium* species [9,11,21,27].

We did not test the detectability of pure sexual parasite stages and thus we cannot address the question of whether the LAMP method is suitable for detection of gametocyte carriers with the goal of malaria elimination. This question needs to be addressed by further studies, while authors have reported interesting results [7].

The highest benefit of this test is its negative predictive value. In emergency situations, this LAMP method will help to exclude patients inadequately suspected of malaria without any more requirements for expert microscopic examination of stained smears. In non-endemic areas, the positivity rate of suspected malaria is highly dependent on the experience of clinicians on call, and high variations may be encountered. However, it could be speculated that a vast majority of malaria tests remain negative. In our experience, a mean of 85% of the 850 malaria tests performed per year for patients suspected of imported malaria are negative. This means that using the LAMP test will reduce the need for a majority of microscopic and RDTs diagnosis, and thus considerably decrease the burden of Quality Assurance requirements for non-specialized laboratories.

In case of positivity, since the subspecies diagnosis is required to adapt first-line treatment in many countries, microscopic tests will be needed. During the night, or during on call periods, a positive LAMP test could be supplemented by a species-specific RDT, to obtain a non-specialized diagnosis of *falciparum* or non-*falciparum* malaria in less than one hour. This emergency diagnosis can be supplemented with smear examinations on the next day to confirm the diagnosis, the species identification, and to determine parasitemia. It should be noted that parasitemia levels are important in case of high values, over the threshold of 4-5%, which remains relatively uncommon. However, in case of high parasitemia, clinical signs of severity are a more reliable marker of a poor outcome than parasitemia. Treatment failures due to complete resistance to the drug used are very uncommon, while resistance to artemisinin derivatives is now a major issue in South-East Asia. Therefore, the usefulness of measuring parasitemia for treatment follow-up has to be balanced with body temperature and clinical presentation.

The basic cost of this test is high in first analysis (approximately USD 25 per single test). However, this high cost should be considered with the fact that the reader may be provided for free or with a very limited cost compared to PCR machines, and that no other reagents are needed. The test can be performed by a non-specialized technician, and since the result is negative/positive, no medical expertise is needed for interpretation. Since the cost of expert human resources is a major part of the global cost of a biological test, this simplification should be taken into consideration for estimating the real cost of the LAMP malaria test. Based on this consideration, value for money and cost-effectiveness appeared acceptable.

Simplicity, robustness, high negative predictive value, high positive likelihood ratios, and the lack of sophisticated equipment suggest that the method could legitimately add value or replace usual microscopic methods for the diagnosis of malaria in laboratories performing the initial emergency screening for imported malaria cases in non-endemic countries. The clinical utility of the Malaria Plus Lamp test from Meridian, as recently defined [2], should be considered positive.

## Conflict of interest

The Associate Editor of Parasite, Stéphane Picot, is one of the authors of this manuscript. COPE (Committee on Publication Ethics, http://publicationethics.org), to which Parasite adheres, advises special treatment in these cases. In this case, the peer-review process was handled by the Editor-in-Chief.

Meridian Inc. provided the machine and 50 Malaria plus tests for the study, but none of the Meridian staff were involved in the study procedure, data analysis, and manuscript writing.
